# Compressed Air Cure

**Published:** 1866-12

**Authors:** 


COMPRESSED AIR CURE.—The proposed plan of curing disease by breathing compressed air, at present gaining some attention by the empirics and public, we shall publish in our next number an extract from a lecture by Prof. Charles A. Lee, containing some interesting facts and observations concerning it. We have some opinions of our own, but reserve expressing them for some future occasion.

